# Preparation and Properties of Magnesium Phosphate Cement-Based Fire Retardant Coating for Steel

**DOI:** 10.3390/ma15124134

**Published:** 2022-06-10

**Authors:** Xiaobing Dai, Jueshi Qian, Jihui Qin, Yanfei Yue, Yushan Zhao, Xingwen Jia

**Affiliations:** College of Materials Science and Engineering, Chongqing University, Chongqing 400044, China; daixb@cqu.edu.cn (X.D.); qinjihui@cqu.edu.cn (J.Q.); yanfei.yue@cqu.edu.cn (Y.Y.); zhaoyushan@cqu.edu.cn (Y.Z.)

**Keywords:** magnesium phosphate cement (MPC), fire-retardant coating, fireproof, expanded vermiculite (EV), corrosion resistance

## Abstract

Magnesium phosphate cement (MPC) is a potential inorganic binder for steel coating due to setting and hardening rapidly, and bonding tightly with steel. NH_4_H_2_PO_4_-based MPC as a fire-retardant coating for steel was investigated in this work. MPC coatings were prepared from MPC paste and MPC mortar with expanded vermiculite (EV). The physical-mechanical properties and fireproof performance of MPC coatings were investigated in detail. An infrared thermal imager was employed to collect the temperature distribution and temperature rise with time on the coating samples automatically. The X-ray diffraction (XRD) and Scanning Electron Microscope (SEM) analyses were carried out on the MPC coating after the fireproof test. Re-fire test and corrosion resistance were performed preliminarily on the MPC coating. The results showed that the fireproof performance of MPC coating met the fire protection requirement for steel as long as the thickness of the MPC paste coating was up to 10 mm, while the thickness of MPC mortar coating decreased to 4 mm when adding 40% EV (by mass). Dehydration and decomposition of reacted products in the hardened MPC coating were, to some extent, contributed to the excellent fireproof performance during the fire test. The slight ceramic formation and integration of MPC coating during the fire test would compensate for the decreasing of strength due to the dehydration and decomposition, so that the MPC coating would keep certain fireproof performance when undergoing fire again. MPC is suitable for a fire-retardant coating, while higher tensile bonding strength with steel and potential corrosion resistance on steel, as well as rapid surface drying and hardening can be achieved.

## 1. Introduction

The steel structure is an excellent construction form, and is used widely in modern architecture due to its lightweight, high strength and green environmental production [1,2,3,4]. To ensure an excellent fireproof performance is essential for the wide use of the steel structure in buildings and other infrastructures; therefore, the fire-retardant coating on the surface of the steel structures is necessary [5]. There are organic and inorganic coatings. The organic coating is usually thin and can exhibit effectively insulating heat and fire due to swelling to 100 times of the original thickness when exposed to fire [6]. However, the organic coating will release a large amount of toxic gas during the preparation, as well as in case of a high temperature [7]. Even if water-soluble solvents are used, the toxic gas release would be avoided in ambient temperature, but not in the case of fire. Therefore, the inorganic coating has attracted more and more attention. Although the organic coating is still dominant in practice, more and more research on inorganic coating is to be carried out [8,9,10,11]. Additionally, some commercial inorganic coatings have been presented.

Inorganic fire-retardant coatings are environmentally friendly, cost-effective and non-combustible, but their properties need to be improved. In general, an inorganic coating would adopt an inorganic binder, such as the ordinary Portland cement (OPC), sulphoaluminate cement (SAC) and magnesium oxychloride cement (MOC) [9,10,12]. Compared with organic binders, these types of cement only develop relatively low bond strength with steel. Therefore, the inorganic coating is easy to break or separate from the steel, especially under the dry-wet cycle. The inorganic coating is usually used in the form of a thick layer of 7 mm to 45 mm. In order to reduce the thickness of the inorganic coating, a lightweight aggregate is usually added to the coating by means of increasing the isolating thermal ability [13]. As a result, the strength of the coating and the bonding strength between coating and steel would decrease sharply. Sometimes, even a protection board would be installed over the coating to avoid it being destroyed. These disadvantages limit to some extent the application of the inorganic coating in practical engineering. It is necessary to develop a potential binder for the inorganic coating of steel structures.

Magnesium phosphate cement (MPC) shows many special properties, such as rapid hardening [14,15], high early strength development [16,17,18], excellent bonding strength [19], good volume stability and outstanding resistance to high temperature [20,21,22,23]. In particular, MPC prefers to bond ferrous materials such as steel, and can protect steel from corrosion [24]. The bond between MPC and steel has been proven to come from both physical and chemical effects. The chemical effect means MPC would react with steel, which provides good bonding between MPC and steel. The dead-burnt magnesia (MgO), constituting the main component of MPC, has a high infrared thermal reflection coefficient [25]. Therefore, MPC is very suitable to be used as the inorganic binder of a fireproof coating of steel. Some researchers have investigated to use MPC as the binder of fireproof coating for steel structures. It can be concluded from their studies that MPC is extremely potential to use as the binder for coating. Almost without exception, however, these investigations selected the potassium dihydrogen phosphate (KH_2_PO_4_) as the acidic component of MPC [23]. MPC can be classified into two types according to phosphate: NH_4_H_2_PO_4_-based MPC and KH_2_PO_4_-based MPC. A lot of previous experimental results confirmed that almost all properties of NH_4_H_2_PO_4_-based MPC are better than those of KH_2_PO_4_-based MPC, except controlling the setting time [20,24]. The hardened NH_4_H_2_PO_4_-based MPC has a higher strength, easily reaching up to 100 MPa in 1 day. It also has lower shrinkage, less than one in ten thousand after the stable state. Most notably, the cost of NH_4_H_2_PO_4_-based MPC is almost half that of KH_2_PO_4_-based MPC. Previous studies avoided using NH_4_H_2_PO_4_-based MPC as the binder, mainly due to worrying about the ammonia release. However, the reaction of NH_4_H_2_PO_4_-based MPC after adding water is as follows:

(1) $${\mathrm{MgO}+{\mathrm{NH}}_{4}H}_{2}{\mathrm{PO}}_{4}{+5H}_{2}O\;\to {\;{\mathrm{MgNH}}_{4}\mathrm{PO}}_{4}{\cdot 6H}_{2}O\;(\mathrm{struvite})\;$$

No ammonia releases in the above reaction, and the product MgNH_4_PO_4_·6H_2_O (struvite, existed stably in nature) is relatively stable when the temperature is below 80 °C and more [17]. Obviously, ammonia is not an intrinsic reaction product when NH_4_H_2_PO_4_-based MPC reacts with water. A qualified NH_4_H_2_PO_4_ product will not release the ammonia when stored and used to prepare MPC. For a coating with a thickness of less than 20 mm, the ammonia release of NH_4_H_2_PO_4_-based MPC is negligible during the preparation and under service conditions. The product MgNH_4_PO_4_·6H_2_O will decompose to generate ammonia in case of fire, which can absorb a lot of heat and isolate the fire. In fact, an ammonia-bearing component is added deliberately to some commercial organic fireproof coatings in order to generate ammonia in case of fire [26]. It can be considered that the ammonia release is conducive to fireproof in case of fire. Therefore, NH_4_H_2_PO_4_-based MPC is much suitable for the binder of inorganic coating. So far, no detailed investigation on using NH_4_H_2_PO_4_-based MPC as the fireproof coating has been reported.

This paper will focus on using NH_4_H_2_PO_4_-based MPC as the binder of an inorganic fire-retardant coating for steel, and compare it with a commercial inorganic fire retardant coating. This study will use a thermal infrared imager to record the temperature distribution and variation of the whole coating at different positions during the fire test, and obtain temperature-time curves. In addition, this study will investigate the early performance of MPC-based coating and the bond strength with steel, to demonstrate the advantage of MPC as the binder of the coating. The fireproof performance of MPC with and without lightweight aggregate EV will be measured. X-ray diffractometer analysis (XRD) and scanning electron microscope (SEM) will be used to characterize the phase evolution and microstructure of the MPC before and after the fire test.

## 2. Materials and Methods

### 2.1. Raw Materials

NH_4_H_2_PO_4_-based MPC was prepared from dead-burnt magnesia (M), ammonium dihydrogen phosphate (P) and borax (B) in the laboratory. The purity of dead-burnt magnesia was about 91% and the particle size of dead-burnt magnesia was less than 75 μm. Both NH_4_H_2_PO_4_ and Na_2_B_4_O_7_·10H_2_O were industrial grade, and the purity was more than 95%. Expanded vermiculite (EV) with an open-pore structure and maximum particle size of 1 mm was used as the lightweight aggregate. The original Q235 steel plate, which accords with GB/T 700 [27] and is widely used in engineering in China, was adopted. A commercial inorganic coating was used for comparison. Ordinary Portland cement (OPC) was selected to be another binder compared with MPC.

### 2.2. Sample Preparation

MPC paste coating and MPC mortar coating with EV aggregate were used to prepare samples in this work. For MPC paste and MPC mortar, the mass ratio of M/P and B/M was fixed at 2 and 0.1, respectively. The composition and main properties of MPC paste are shown in Table 1. Due to the large water absorption of EV, the added water should be adjusted according to the amount of EV to obtain appropriate workability as adding EV, and the added water was twice that of EV. Ratios of water to cement were 0.40, 0.80 and 1.20 when the content of EV aggregate were 20%, 40% and 60% of the cement (by mass), named M20, M40 and M60, respectively. For the commercial coating, a water-mixture ratio of 0.75 suggested by the supplier was used. All mixtures were pre-mixed for 2.5 min at a low speed, and then mixed for 3 min at a high speed after adding water. All mixture was kept as similar fluidity as possible. OPC paste was prepared using a water-to-cement of 0.25 and polycarboxylate superplasticizer-to-cement of 1‰ to obtain a compressive strength of 65 MPa at 28 days.

Specimens for fire test consist of 140 × 140 × x (thickness x = 4, 6, 10, 14) mm^3^ coating and 140 × 140 × 2 mm^3^ steel plate as the base plate. The MPC coating specimens for the fire test were made of self-made mould. The mould was made by steel plate (150 × 150 × 2 mm^3^) and double-sized tape of certain thickness. The tape with certain thickness (thickness x = 4, 6, 10, 14 mm) was fixed to the edge of the steel plate tightly, and then the MPC was casted into the self-made mold. Specimens for the tensile bonding strength between coating and steel plate, consist of 40 × 40 × 10 mm^3^ coating and 70 × 70 × 6 mm^3^ steel plate as the base plate. Cubic specimens with a side length of 25 mm were used to measure the compressive strength and bulk density, and a thin slice of 10 × 10 × 1 mm^3^ to measure the thermal conductivity.

All specimens made of MPC mixture were removed from the moulds after 1 h, and placed in the laboratory for air curing at 20 ± 1 °C and 65% relative humidity (RH). The age of MPC paste and mortar coatings for the fire test was 7 days, unless otherwise specified. The specimen made of the commercial coating was cured for at least 7 days before being removed from the moulds, and underwent the fire test at 28 days.

The crushed samples of MPC paste coating were immersed in an excess of absolute ethanol to stop the further reactions. After immersed for 24 h, samples were dried at 40 °C for 24 h in a vacuum desiccator before performing X-ray diffraction (XRD) (Panalytical, Almelo, The Netherlands) and scanning electron microscopy (SEM) (TDK, Tokyo, Japan) analysis. The samples for SEM analysis were sputtered with a gold coating before the test.

### 2.3. Test Methods

#### 2.3.1. Fire Test

An alcohol blast burner with a maximum temperature of 950 °C was used as a heat source. The alcohol consumption rate of the burner was kept at 150–170 g/h. Two burners were shifted interchangeably, since an alcohol blast burner can last for half an hour. The surface coated by MPC of the specimen was exposed directly to the flame of the alcohol blast burner. The vertical distance between the top of the alcohol blast burner and the bottom of the specimen was 70 mm.

**Figure 1:**
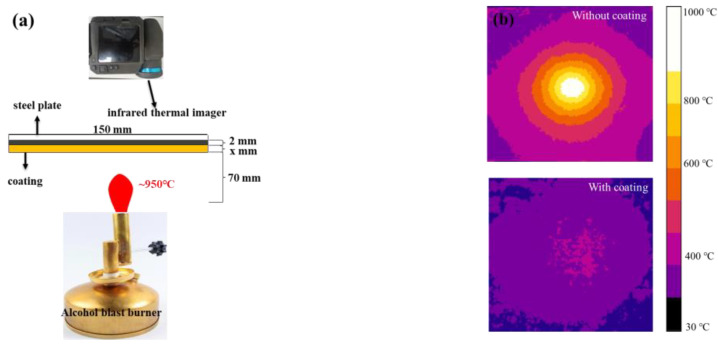
Schematic diagram of fire test: (**a**) Assembled system; and (**b**) Infrared thermal images of the back of the steel plates without and with coating.

The temperature distribution on the back of the steel plate was measured continuously by a thermal infrared imager (FLIR T540) (FLIR Systems, Boston, MA, USA) with a temperature measurement range of −20 to 1200 °C. The thermal infrared imager can record and store the temperature distribution with time automatically, and pick out the maximum temperature at any moment during the fire test. The temperature rise curve at any point of the steel plate was plotted. Considering the maximum temperature point drifted at different moments, the temperature rise curves in the center point of the steel plate were given in this work. The system assembled for the fire test is shown in Figure 1a. The temperature distribution on the back of a steel plate with or without coating is shown in Figure 1b.

#### 2.3.2. Physical-Mechanical Properties of the Coating

Setting time was determined by Vicat needle according to ASTM standard C191 [28]. Since the time interval between the initial and final setting is very short, only the final setting time value is presented in this paper. The compressive strength was measured using a compression machine with a maximum loading of 150 kN and a loading speed of 0.5 kN/s. Three specimens were used to test the compressive strength, and then the average was taken.

**Figure 2:**
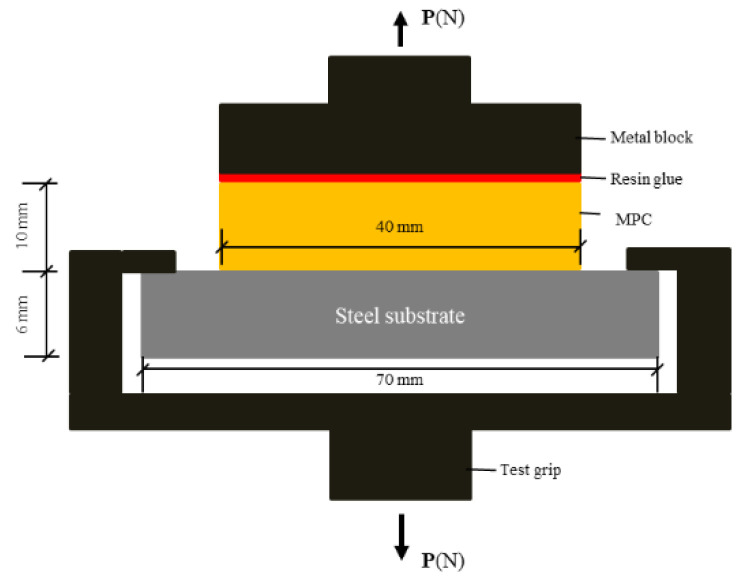
The setup for tensile bond strength of MPC.

The tensile bonding strength between the coating and steel plate was determined according to ASTM D 4541 (Figure 2). Five specimens were used to test the tensile bonding strength and then the average was taken. The effect of dry-wet cycles on the tensile bonding strength between coating and steel plate was investigated. The coating was cured in the air for 1 day before the dry-wet cycle. A dry-wet cycle was soaked in water at 20 °C for 12 h, and then moved to an oven at 40 °C for another 12 h.

The surface drying time was determined according to the finger-touching method specified by Chinese Standard GB/T 13477.5-2002 [29]. Bulk density was tested according to Chinese Standard GB/T 24586-2009 [30].

The thermal conductivity of the coating was measured using a laser thermal conductivity meter at 20 °C in a nitrogen atmosphere. The samples were dried for 1 day in a 40 °C oven before testing.

#### 2.3.3. X-ray Diffraction

The hydration production and phase evolution of MPC paste coating were identified using X-ray diffraction (Copper anode λKα1 = 1.54056 Å generated at 40 mA and 40 kV). The samples were ground to a particle size less than 75 μm. The acquisition range was from 5° to 65° at 0.02° steps with integration at the rate of 32 s per step.

#### 2.3.4. Scanning Electron Microscopy

The morphological characteristics of MPC paste and mortar coatings before and after the fire test were observed using a JEOL JSM-7800F filed-emission scanning electron microscopy coupled with an energy dispersive spectroscope (EDS). All SEM images were acquired at an accelerating voltage of 10–15 kV and a working distance of about 10 mm.

#### 2.3.5. Electrochemical Measurement

The specimens cured for a certain period were subjected to an accelerated corrosion test. A dry-wet cycle of the accelerated corrosion was carried out by drying at 40 °C for 12 h in an oven and then immersing in 3.5 wt.% NaCl solution for 12 h. The corrosion current density was measured using an electrochemical workstation (Zahner, Krona, Germany) after each dry-wet cycle before first 7 cycles and then every two cycles. Electrochemical measurements were performed using the typical three-electrode system, which including a saturated calomel electrode as a reference electrode, a platinum sheet (25 × 25 × 0.2 mm^3^) as a counter electrode and the specimens as working electrodes. After 16 cycles of accelerated corrosion test, the surface state of steel base was observed after removing the coating.

## 3. Results and Discussion

### 3.1. Basic Properties of MPC Paste Coating

The setting time and strengths of MPC paste were listed in Table 1. It is worth noting that the optimal ratio of water to MPC is about 0.12–0.15 [14], but a water to MPC ratio of 0.20 was used in this work due to the subsequent addition of the lightweight aggregate, which would require a higher ratio. The compressive strength of MPC paste at 3 h was 25.2 MPa, and more than 60 MPa at 1 day.

**Figure 3:**
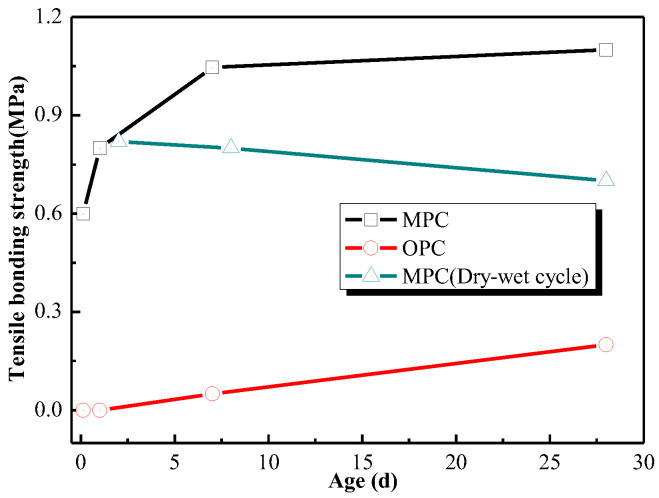
The tensile bonding strengths of MPC and OPC with steel plate.

As a coating, it is of important to keep enough bonding strength with a substrate. The MPC can get a certain bonding strength with steel in a short time. Figure 3 shows the tensile bonding strengths of MPC coating and OPC-based coating with steel. It can be seen from Figure 3 that MPC coating could get 0.60 MPa bonding strength at early 3 h, and more than 1.0 MPa at 7 days, while OPC coating rarely produced any strength at 1 day and only 0.21 MPa boning strength at 28 days. The superior interfacial bond strength between MPC and steel comes from both physical and chemical effects. From the chemical effect perspective, the acidic phosphate contained in MPC would react with steel at the early age, which may benefit to strong bond between MPC and steel plate. For the physical effects, the shrinkage of MPC was much lower than that of OPC [14], which can reduce occurrence of micro-cracking on the interface and thus enhance the bond strength. In order to investigate the stability of MPC coating bonding with steel, a dry-wet cycle test was performed on the coating. The tensile bonding strength of MPC coating after one cycling was about 0.8 MPa, and still maintained about 0.75 MPa after 27 cycles. It is shown that the MPC exhibited a higher bonding strength far higher than 0.04 MPa required by Chinese standard GB14907-2018 [31].

### 3.2. Fireproof Performance of MPC Paste Coating

**Figure 4:**
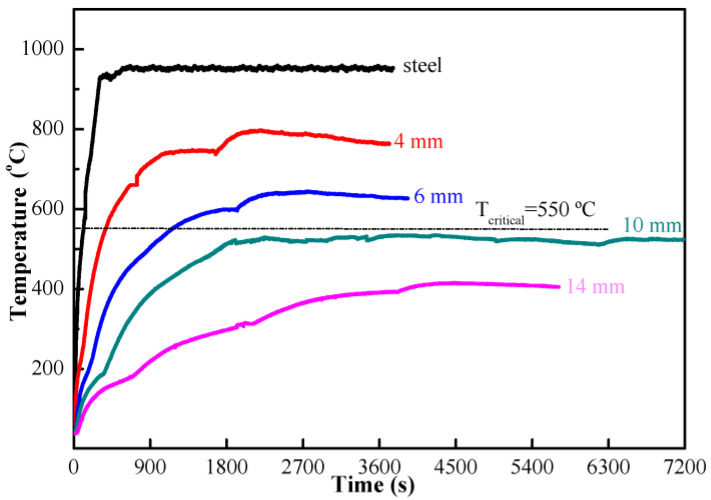
Temperature rise curves of NH_4_H_2_PO_4_-based MPC coating.

Figure 4 shows the temperature rise curves of steel plates with and without MPC coating. The thickness of MPC paste coating varies from 4 mm to 14 mm (Figure 4). It is shown from Figure 4 that the temperature rise of MPC coating can meet the critical temperature (550 °C) of GB 14907-2018 (dotted line in Figure 4) [31], as long as the thickness of MPC coating is equal to or more than 10 mm. The temperature of the steel plate without MPC coating rose over 900 °C after fired for 300 s. The maximum temperature of the steel plate with MPC coating appeared much later than that of the steel plate. With the increase of coating thickness, the maximum temperatures reduced sharply and the time when the maximum temperature appeared prolonged. For the steel plate with a 10 mm MPC coating, the maximum temperature was 524 °C and appeared at more than 2400 s.

**Figure 5:**
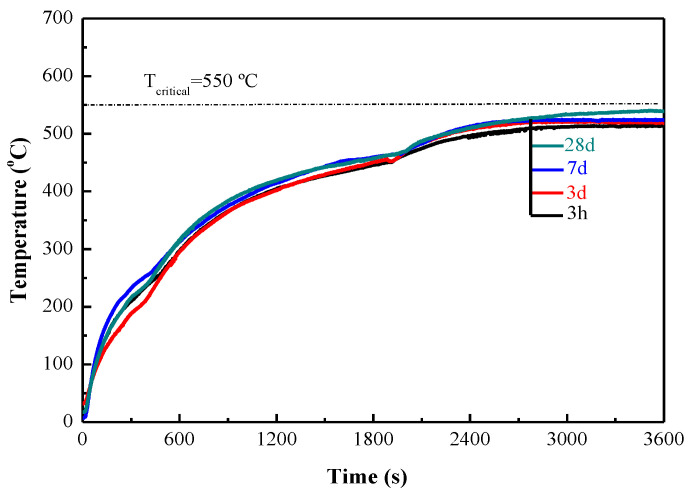
Effect of age on fireproof performance of 10 mm MPC coating.

MPC can set and harden rapidly compared with other binders for coating. Figure 5 shows the fireproof performance of MPC coating with a thickness of 10 mm at ages of 3 h, 3 days, 7 days and 28 days, respectively. The temperature rise curves of MPC coating behaved the same characteristic from 3 h to 28 days. As shown in Figure 5, MPC coating can take effect as early as at least 3 h, and the fireproof performance did not vary significantly with age.

It is shown from the above experimental results that MPC coating has an excellent fireproof performance, especially at an early age of 3 h. The MPC coating with a thickness of 10 mm can meet the requirement of the highest temperature of less than 550 °C and exhibit high strength at early age of 3 h. It is worth noting that the curves do not exhibit smooth, since the temperatures at the center of the sample are recorded automatically by the thermal infrared imager and the burner is shifted every about 30 min.

### 3.3. Fireproof Performance of MPC Mortar Coating with EV

Sometimes, reducing the thickness of a coating is of particular concern. To add lightweight aggregate into the binder is a common method.

**Figure 6:**
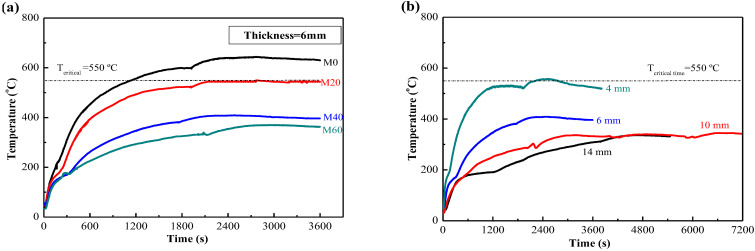
Temperature rise curves of MPC with EV: (**a**) Effect of EV content in MPC coating on the maximum temperature; and (**b**) Effect of thickness of MPC coating with 40% EV on the maximum temperature.

Figure 6a shows the temperature rise curves of MPC mortar coating with the same thickness of 6 mm when the mass ratio of EV as lightweight aggregate to MPC increased from 0% (M0) to 60% (M60). It is shown that the fireproof performance of MPC mortar with EV can meet the requirement of the highest temperature of less than 550 °C, as long as EV content is 20% (M20) and coating thickness is 6 mm.

The coating thickness would be reduced further when more EV is added into the coating. Figure 6b shows the temperature rise curves of MPC mortar coating with different thicknesses when the mass ratio of EV to MPC was 40% (M40). It is shown that the fireproof performance of MPC mortar with 40% EV can meet the requirement of the highest temperature of less than 550 °C, as long as coating thickness is 4 mm. Furthermore, the maximum temperature was about 550 °C, and appeared at more than 2400 s. It is generally accepted that the thickness of less than 7 mm can be classified as a thin coating [31], thus MPC mortar with a certain amount of lightweight aggregate can meet the requirement of a thin fireproof coating.

**Figure 7:**
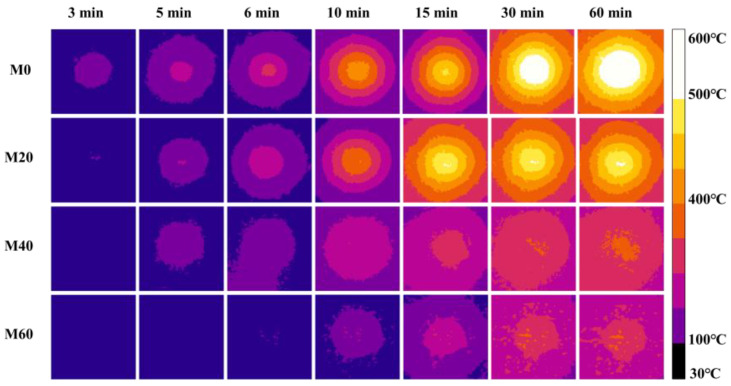
Infrared thermal image of MPC with varying EV content in 6 mm.

Figure 7 shows the infrared thermal image of MPC coating with different EV contents at different firing times. It is shown that the temperature at the same firing time would decrease markedly, and the maximum temperature appeared late with the increase of EV content. The water consumption of MPC mortar increased with the increase of EV content, resulting in a higher porosity of the matrix within MPC mortar and thus a lower thermal conductivity in comparison to the pure MPC matrix. On the other hand, the addition of EV containing porous lamellar structure could enhance the thermal insulation capacity of MPC mortar, thereby lowering the temperature [13].

**Figure 8:**
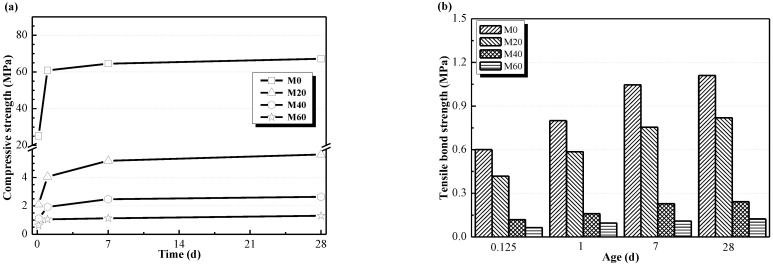
Strengths of MPC mortar with different EV contents: (**a**) compressive strength; and (**b**) tensile bonding strength.

The addition of lightweight aggregates such as EV would definitely reduce the strength of the coating. Figure 8 shows the compressive strength of the coating and tensile bonding strength between the coating and steel plate decreased with the EV content increased. When the EV content was 20% (M20), all strengths would reduce significantly. However, even EV content was 60% (M60), the strength would meet the compressive strength of more than 0.3 MPa and the tensile bonding strength of more than 0.04 MPa specified by Chinese standard GB 14907-2018 [31].

**Figure 9:**
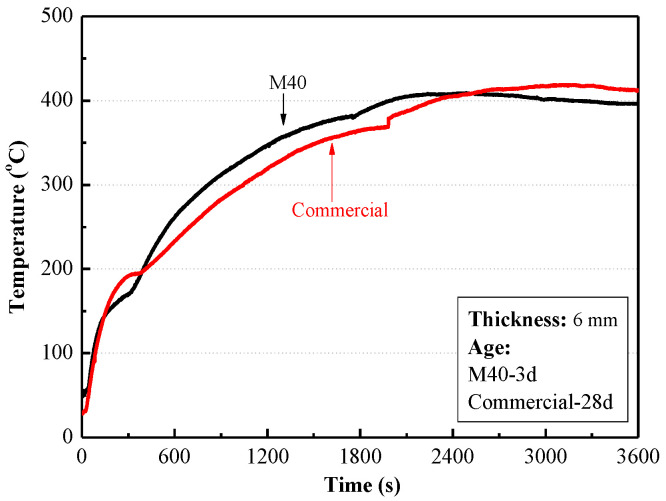
Temperature rise curves of MPC mortar coating with 40% EV and a commercial inorganic coating.

Table 2 and Figure 9 show comparison of main properties of MPC mortar coating with 40% EV and a commercial inorganic coating. The thickness of all coatings was 6 mm. It can be seen from Table 2 and Figure 9 that MPC mortar coating with 40% EV can achieve similar fireproof performance to the commercial inorganic coating, but higher strengths and a shorter drying time. It can be concluded that the thickness of MPC coating can be reduced significantly by adding lightweight aggregate.

### 3.4. Phase Evolution and Microstructure Change of MPC Coating after Fire Test

The microstructure and reacted products of the MPC coating would change during and after the fire test. Dehydration of the products is the main reaction, and new minerals will occur during the fire test. It is concerned that the dehydration and formation of new minerals would not significantly affect the mechanical properties of the MPC coating.

**Figure 10:**
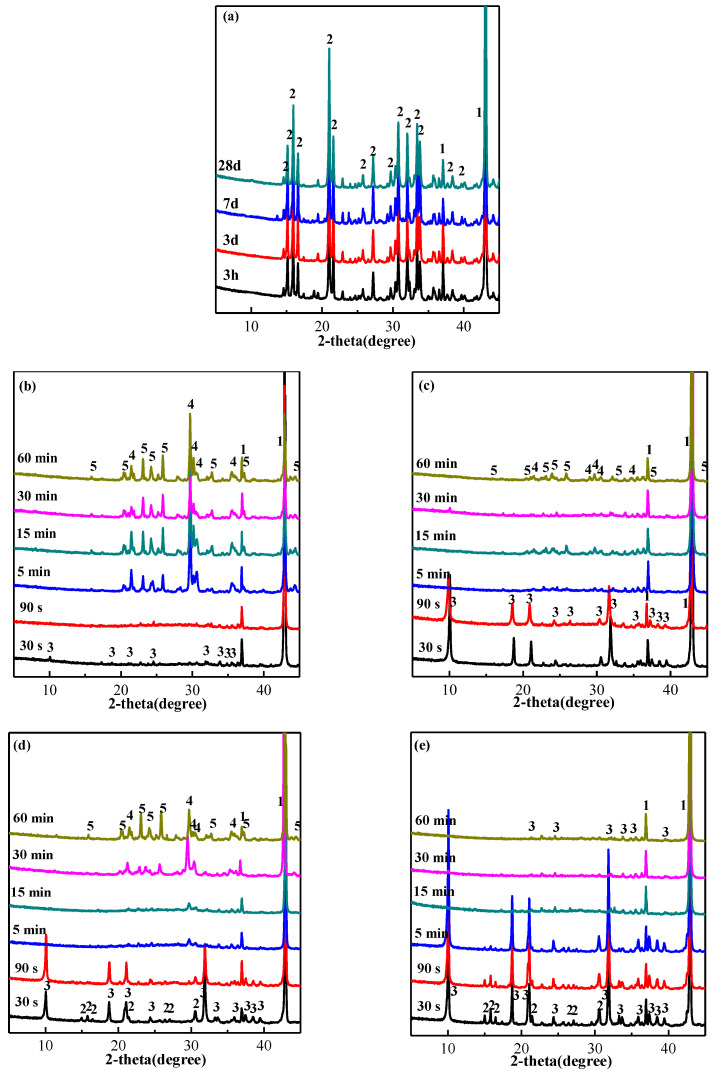
XRD pattern of MPC (**a**) original MPC coating at different ages; (**b**) the surface of 4 mm coating fired; (**c**) the interface of 4 mm coating fired; (**d**) the surface of 14 mm coating fired; and (**e**) the interface of 14 mm coating fired. (1 = MgO, 2 = MgNH_4_PO_4_·6H_2_O, 3 = MgNH_4_PO_4_·H_2_O, 4 = Mg_2_P_2_O_7_, 5 = Mg_3_(PO_4_)_2_.).

Figure 10 shows the XRD patterns of the hardened NH_4_H_2_PO_4_-based MPC coatings from 3 h to 28 days, and those of MPC coatings fired for from 30 s to 60 min. The samples were obtained from the surface of the coating and the interface between the MPC coating and steel. The age of MPC coatings was 7 days, and their thicknesses varied from 4 mm to 14 mm. It can be seen that struvite, the main hydration product on the surface of 4 mm coating, was dehydrated into dittmarite after fired for 30 s, while that of 14 mm coating was when fired for 5 min. The hydration product on the surface of the 4 mm coating was partly dehydrated into magnesium phosphate and farringtonite when fired for 15 min. The dehydration rate slowed down with the increase of coating thickness. There was still a small amount of struvite in the surface of 14 mm coating when fired for 30 s, and no magnesium phosphate and farringtonite in the interface when fired for 15 min. The phase evolution from struvite to dittmarite, magnesium phosphate and farringtonite would reduce the strength of coating [20], and the residual strength depended on the temperature. When the temperature was between 800 and 900 °C the residual strength was lowest, but more than 30% of the original [23]. When the temperature was above 900 °C, the residual strength increased, and even the strength was higher than the original strength when the temperature was above 1000 °C due to some ceramic formation in the coating [32]. During the fire test, the mineral compositions of the hardened MPC coating changed not only with the time fired, but also with the position. As the time fired prolonged, the hydration product struvite was dehydrated into dittmarite, then dehydrated further into magnesium phosphate, and finally calcinated into farringtonite, a kind of ceramic formed the reaction between magnesium phosphate and the excessive MgO in MPC coating. Additionally, the mineral compositions varied across the coating. For the 14 mm coating, farringtonite began to form on the surface after fired 30 min, but only dittmarite existed in the interface between the coating and steel when fired for 60 min.

**Figure 11:**
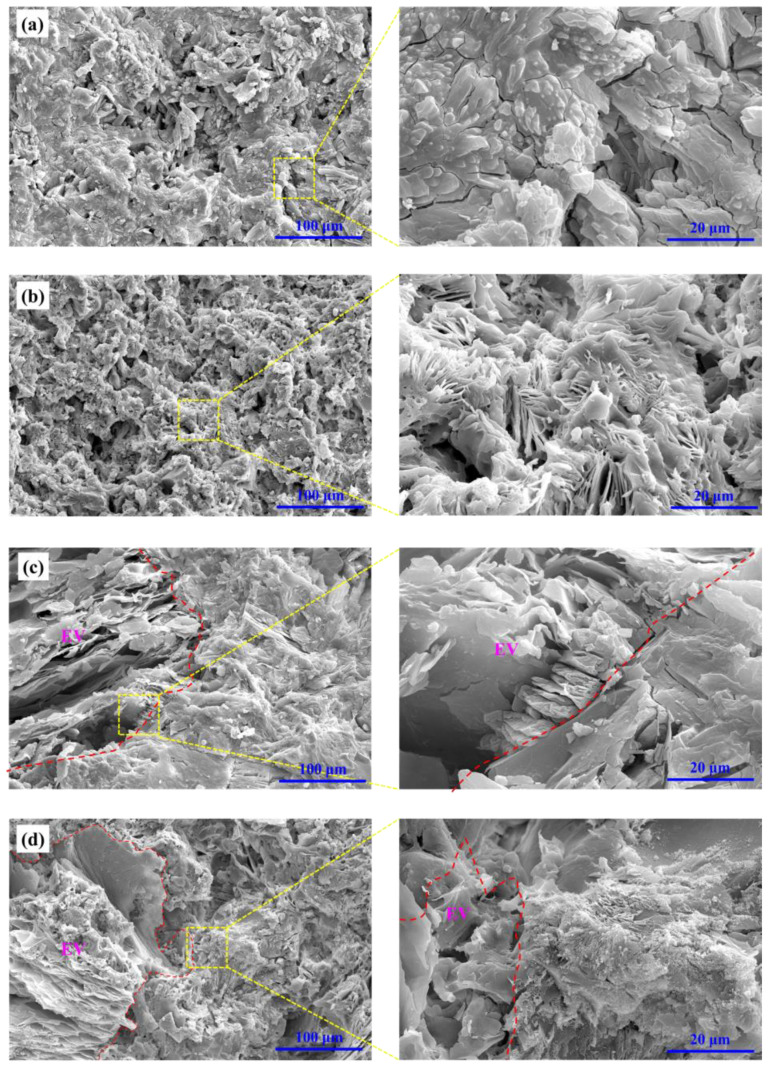
SEM image of the fracture surface for MPC: (**a**) at room temperature; (**b**) after fireproof performance test; SEM image of the fracture surface for MPC with 40% EV: (**c**) at room temperature; and (**d**) after fireproof performance test.

Figure 11 shows the morphology of hydration products formed in MPC coating with and without EV before and after the fire test. The age of MPC coatings was 7 days, and the fire test lasted 3600 s. It can be seen that the MPC coating became more porous, but micro-cracks almost disappeared after the fire test, due to the decomposing of the hydration product and the ceramic formation. In addition, the lightweight aggregate EV was integrated with the MPC matrix after the fire test. Because the flame temperature of the alcohol blast burner reached up to 950 °C, no sintering occurred in the slightly ceramic formation, resulting in no shrinkage of the coating after the fire test. It can be concluded that the MPC coating would keep a certain strength and integration after firing, so it can be used for permanent fire protection.

### 3.5. Discussion

Table 3 gives the comparison of the main properties of the MPC paste coating, MPC mortar with EV and the commercial inorganic coating.

**Figure 12:**
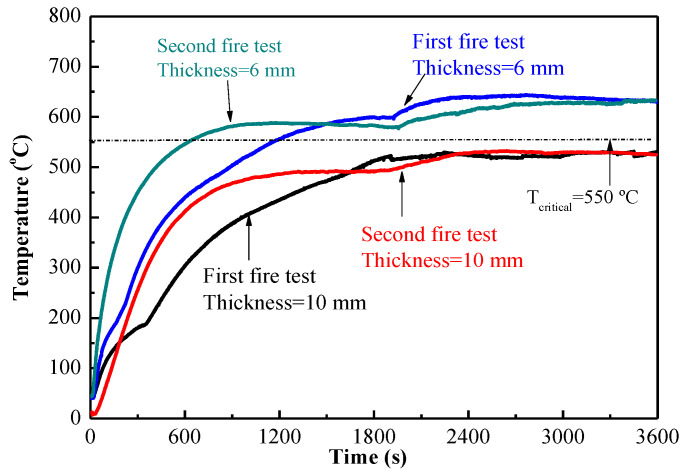
Comparison of temperature rise curves between the first and second fire tests.

MPC paste coating has relatively higher strength, particularly higher tensile bonding strength. In order to meet the requirement of fireproof specified by Chinese standard GB 14907-2018 [31], the thickness of MPC paste coating would be up to 10 mm. The thicker MPC paste coating can be used in some fields, which require higher strength, permanent fireproof ability and anti-corrosive ability. Figure 12 shows the temperature rise of MPC paste coating undergoing the second fire test. Except for the temperature rising quickly due to no dehydration, the maximum temperature of the second fire test is similar to that of the first test. Because the MPC coating can form ceramic with the firing and keep relatively higher strength during firing, it can be used as a permanent fireproof coating.

**Figure 13:**
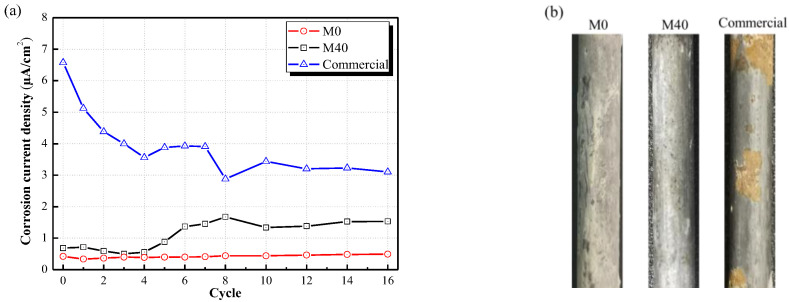
Corrosion resistance of MPC paste coating, MPC mortar coating and commercial coating (thickness = 8 mm). (**a**) Corrosion current density of different coatings at 16 cycles of accelerated corrosion test; and (**b**) Surface state of base steel plates after 16 cycles of accelerated corrosion test and removing the coatings.

Another disadvantage of steel is poor corrosion resistance. Thicker MPC paste coating can provide a certain anti-corrosive ability. Figure 12 provides the comparison of corrosion resistance of MPC paste coating, MPC mortar coating and the commercial coating with the same thickness of 8 mm after 16 cycles of accelerated corrosion test. The age of MPC coatings was 7 days and the commercial coating was 28 days. It can be seen from Figure 12 that MPC paste coating and MPC mortar coating with 40% EV could protect steel from corrosion. The corrosion current density of MPC paste coating was still below 0.50 μA/cm^2^, and even that of MPC mortar coating with 40% EV could be below 2.0 μA/cm^2^. However, the corrosion current density of the commercial coating had been in a relatively high range, and the steel base exhibited relatively severe rust (Figure 13b). The corrosion resistance of MPC coating will be investigated further, especially regarding MPC mortar coating with lightweight aggregate.

It can be seen from Table 3 that the thickness of MPC coating was reduced by adding lightweight aggregate EV. Compared with the commercial coating, MPC coating also can be used in a thin layer of 4 mm when 40% EV is added. The paste coating and MPC mortar coating would be employed in different conditions. In the case of requiring high strength and fireproof performance of the coating, MPC paste coating would be the best choice, while MPC mortar coating for requiring fireproof performance, thinner and lighter coating. The MPC paste coating not only had high early strength, compressive strength of 25.2 MPa at 3 h, but also had high strength, 64.7 MPa at 1 day and 67.2 MPa at 28 days (see Table 1 and Table 3). In fact, MPC paste coating can achieve very high compressive strength of over 100 MPa. The addition of lightweight aggregate would decrease the strength of MPC mortar coating, but it can be seen from Table 3 that the compressive and tensile bonding strengths were 1.10 MPa and 0.11 MPa at 3 h, and 2.3 MPa and 0.24 MPa at 28 days, respectively, when 40% EV was added. Even the strengths at 3 h were far higher than that required by the standard or achieved by the commercial coating at 28 days. The strengths of MPC mortar coating with 40% EV are enough not to provide an extra protective layer above. The durability of the coating with EV will be systematically investigated in the future.

## 4. Conclusions

This paper aimed to develop a fire-retardant coating for steel based on MPC, and understand the fireproof performance of the coating. The conclusions are summarized below:

(1) NH_4_H_2_PO_4_-based MPC can be used as the binder of inorganic fire-retardant coating for steel. The surface drying time of coating was less than 30 min and the fireproof performance of coating took effect as early as 3 h. The tensile bonding strength between MPC coating and steel was 0.6 MPa at 3 h and 1.10 MPa at 28 days, far higher than that required by Chinese standard. It illustrated that the MPC coating not only set rapidly, but also bound tightly to steel.
(2) The fireproof performance of MPC paste coating met the requirement, the maximum temperature being less than 550 °C after firing for 2 h, as long as the thickness was up to 10 mm, while the thickness of MPC mortar coating decreased to 4 mm when adding 40% expanded vermiculite (EV) as a lightweight aggregate. MPC coating with and without lightweight aggregate can be used in different conditions.
(3) Dehydration and decomposition of the reacted products in MPC coating during the fire test are good for the fireproof performance of MPC coatings. The slight ceramic formation of MPC coating during the fire test would compensate for the decrease of strength and shrinkage of volume. The fireproof performance of MPC coating in the second fire test was similar to that in the first fire test. MPC coating may be even potential to use for permanent fireproof conditions.
(4) Considering the comprehensive performance but not the only fireproof performance of MPC coatings, including high tensile bonding strength with steel, rapid hardening, excellent corrosion resistance to steel and permanent fireproof performance, MPC coating for steel exhibits more advantages. Further research or improvement on this coating is necessary.

## Figures and Tables

**Table 1:** Composition and main properties of MPC paste.

Composition	Magnesia (MgO, M)	62.5% (wt)
	Ammonium dihydrogen phosphate (NH_4_H_2_PO_4_, P)	31.3% (wt)
	Borax (Na_2_B_4_O_7_·10H_2_O, B)	6.2% (wt)
	Water-cement ratio (W/C)	0.20
Setting time (min)		26.0
3 h compressive strength (MPa)		25.2
1 day compressive strength (MPa)		60.9
7 days compressive strength (MPa)		64.5
7 days flexural strength (MPa)		7.2

**Table 2:** Comparison of main properties of MPC mortar coating with 40% EV with a commercial inorganic coating.

	Age	Compressive Strength (MPa)	Tensile Bonding Strength (MPa)	Surface Drying Time (min)	Bulk Density (g/cm^3^)	Thermal Conductivity (W/(m·K)
Commercial	28 days	0.4	0.06	750	0.71	0.15
MPC mortar with 40% EV	3 h	1.1	0.11	25.5	0.79	0.26
	28 days	2.6	0.24			

**Table 3:** Main properties of MPC paste and mortar coating.

Type of Coating	MPC Paste Coating	MPC Mortar Coating with EV (mass)			Commercial Coating
		20% EV	40% EV	60% EV	
Surface drying time (min)	23.3	28.1	25.5	26.9	750
Thermal conductivity (W/(m·K))	2.11	0.41	0.26	0.18	0.15
Bulk density (g/cm^3^) (Dry)	2.16	1.42	0.78	0.61	0.71
3 h Compressive strength (MPa)	25.2	2.10	1.10	0.63	n.d.
3 h Tensile bonding strength (MPa)	0.60	0.42	0.11	0.06	n.d.
28 d Compressive strength (MPa)	67.2	5.62	2.40	1.30	0.4
28 d Tensile bonding strength (MPa)	1.11	0.82	0.24	0.12	0.08
Minimum thickness of coating (mm)	10	6	4	<4	4

n.d.: Not detected.

## Data Availability

The data presented in this study are available on request from the corresponding author.
